# Long‐term trends in sex difference in bladder cancer survival 1975‐2009: A population‐based study in Osaka, Japan

**DOI:** 10.1002/cam4.3382

**Published:** 2020-08-13

**Authors:** Jo Aoe, Yuri Ito, Keisuke Fukui, Masashi Nakayama, Toshitaka Morishima, Isao Miyashiro, Tomotaka Sobue, Tomio Nakayama

**Affiliations:** ^1^ School of Medicine Osaka University Osaka Japan; ^2^ Department of Medical Statistics, Research & Development Center Osaka Medical College Osaka Japan; ^3^ Department of Urology Osaka International Cancer Institute Osaka Japan; ^4^ Cancer Control Center Osaka International Cancer Institute Osaka Japan; ^5^ Division of Environmental Medicine and Population Sciences Graduate School of Medicine Osaka University Osaka Japan; ^6^ Division of Screening Assessment and Management Center for Public Health Sciences National Cancer Center Tokyo Japan

**Keywords:** Cancer registration, Population‐based study, Sex, Survival Analysis, Urinary Bladder Neoplasms

## Abstract

In Japan, a study using population‐based cancer registry data from six prefectures revealed a difference in bladder cancer survival between men and women. However, the period of the study was limited to 1993‐2006. The recent introduction of immune checkpoint inhibitors, which have proved to be effective for the treatment for bladder cancer, has led to a rising demand for analysis of long‐term trends in net survival in order to accurately assess the effect of the new treatment. The aim of the present study was to examine long‐term trends in sex difference in bladder cancer net survival using large‐scale population‐based cancer registry data from Osaka, Japan (17,500 cases from 1975 to 2009). We also evaluated sex difference in bladder cancer survival after adjustment for stage, histologic type, and other prognostic factors. We showed the long‐term trend of five‐year net survival for each stage and found that women had poorer five‐year net survival than men for the whole study period. The risk of death from bladder cancer was higher among men than women even after adjusting for period at diagnosis, histologic type, stage, age group, and treatment (Excess hazard ratios: 1.17; 95% Confidence interval: 1.10‐1.25).

## Graphical abstract

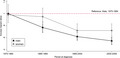

## 1 INTRODUCTION

Bladder cancer, the ninth most frequently diagnosed cancer worldwide, has a lower incidence rate among women.^1^ However, unlike most cancers, bladder cancer has lower survival for women than men in Europe, Australia, and the USA.^2^, ^3^, ^4^, ^5^, ^6^, ^7^ This is, in part, due to women having a higher percentage of non‐urothelial cancer, which has worse survival.^8^ Nevertheless, even after adjustment for histologic type, women still have poorer survival according to a study on white and black Americans.^9^

In Japan, a study based on population‐based cancer registry data from six prefectures revealed a similar sex difference in bladder cancer survival.^10^ This study showed that the cancer stage at initial diagnosis is more advanced in women than in men and women with localized or regional bladder cancer have a worse prognosis than men. However, the period of the study was limited to 1993‐2006.

Recently, immune checkpoint inhibitors have been introduced and have proved to be effective for the treatment of bladder cancer.^11^ As a result, there has been a rising demand for analysis of the long‐term trends in net survival and sex difference among bladder cancer patients in Japan in order to gain accurate knowledge about the effect of the new treatment.

However, to date no research has analyzed these long‐term trends in Japan. The aim of the present study was, therefore, to assess long‐term trends in sex difference in net survival of bladder cancer using large‐scale population‐based data from the Osaka Cancer Registry, Japan. We also evaluated sex difference in bladder cancer survival having adjusted for stage, histologic type, and other prognostic factors.

## 2 MATERIAL AND METHODS

### 2.1 Data sources

We used Osaka Cancer Registry (OCR) data on 19 765 cases which had been diagnosed as primary malignant bladder cancer between 1975 and 2009. Among the largest population‐based data registries worldwide, the OCR holds data on 8.8 million patients. It has been monitoring cancer patients for over 50 years which makes the data particularly useful for the evaluation of long‐term trends in cancer survival. The majority of cases (85.3%) were microscopically verified; 11.5% were death certificate only (DCO). We excluded the DCO cases (11.5%, 2263 cases) and also patients aged over 100 as life tables were not available for them (9 cases, 0.05%), leaving a final total of 17,500 eligible cases for survival analysis. In our analysis, bladder cancer ranges from C67.0 to C67.9 in ICD‐O‐3. Morphological categories range from 8010 to 9120. Clinical stage does not include Ta or Tis.

Year of diagnosis was divided into four periods: 1975‐1984, 1985‐1994, 1995‐2004, and 2005‐2009. Patients who had been diagnosed between 1975 and 2004 were followed up for at least 10 years while those diagnosed between 2005 and 2009 were followed up for at least 5 years. 4.3% cases were lost‐to‐follow‐up within five years. We defined three categories for age at diagnosis: 0‐64 years, 65‐74 years, and 75‐99 years old. We classified histologic type as urothelial or non‐urothelial carcinoma according to the 2016 WHO Classification of Tumors ^12^ and histology codes 8020, and 8120‐8131 of the International Classification of Disease for Oncology version 3 Morphology (ICD‐O‐3 M) were used for urothelial tumors. Stage at diagnosis was classified according to SEER summary stages.^13^ These stages and the corresponding International Union Against Cancer (UICC)^14^ tumor node metastasis (TNM) classifications are: (i) localized: cancer is confined to the organ from which it originated (TNM: T1‐2 N0 M0), (ii) regional metastasis: cancer has metastasized into regional lymph nodes and/or invaded adjacent organs (TNM: T1‐2 N1‐3 M0 or T3‐4 N0‐3 M0), (iii) distant metastasis: cancer has metastasized into distant organs and/or lymph nodes (TNM: T_any_ N_any_ M1). Treatment categories were: surgery, radiotherapy, and chemotherapy and treatment the variables were “performed,” “not performed,” and “unknown.” For the purposes of this analysis, we treated “unknown” as “not performed.” Of the total “unknown” category for surgery, radiotherapy and chemotherapy, the proportions were 0%/5.5%/5.1% for men and 0%/5.6%/5.2% for women, respectively.

### 2.2 Statistical analysis

In each period, five‐year net survival was estimated by histologic type, stage, treatment (surgery, chemotherapy, radiotherapy, and immunotherapy), age group, and sex according to the method introduced by Pohar Perme et al.^15^ Japanese life tables ^16^ were used to control for background mortality. Moreover age‐standardized 5‐year net survival was calculated using the International Cancer Survival Standard Weights.^17^ As in some categories the number of cases was too small for accurate analysis, we used three rather than five age groups for age standardization. Standard 1 weights were categorized into 0.42 for (0‐64), 0.29 for (65‐74), and 0.29 for (75‐99). We used flexible multivariable parametric excess hazard models^18^, ^19^ to consider the effect of prognostic factors: sex, period at diagnosis, histologic type, stage, age group, and treatment. We used the *stns* command to estimate net survival and *stpm2* to apply the flexible multivariable parametric excess hazard model using Stata version 14 (STATA Corporation, College Station, TX, USA) for data management and statistical analysis.

Data on histologic type were missing for 7.9% of cases (We define ICD‐O‐3 code 8000 as missing) and 10.5% did not have stage at diagnosis (for detailed information on characteristics of bladder cancer patients, see Table S1). We assumed these data to be missing at random (MAR) and used multiple imputation (MI) to handle the missing values.^20^ For each incomplete variable (histologic type and stage), we used the ordered logistic regression model including another incomplete variable, complete variables (follow‐up time, vital status [alive or dead] sex, treatment, age at diagnosis, and period at diagnosis) and interactions between follow‐up time and other variables. MI yielded 10 complete data sets with imputed values for the missing variables. Finally, we used Rubin's rule to calculate net survival, EHRs, and standard errors combining the 10 imputed data sets.^21^

## 3 RESULTS

We found that the proportion of urothelial carcinoma (UC) in bladder cancer patients did not change much among either women or men. The proportion of patients aged 75‐99 years increased from 30.0% to 52.1% in women and from 23.8% to 38.8% in men, indicating an increase in age at diagnosis. The percentage of men diagnosed at a distant stage decreased from 9.4% in 1975‐84 to 5.5% in 2005‐09, while the percentage of women diagnosed at a distant stage decreased from 12.1% in 1975‐84 to 6.3% in 2005‐09. During 1975‐84, the difference was approximately 2.7% (12.1% in women and 9.4 in men); in 2005‐09 it decreased to 0.8% (6.3% in women and 5.5 in men) (Table 1).

**Table 1:** Characteristics of bladder cancer patients in Osaka, Japan stratified by sex, period at diagnosis, histologic type, stage, age group, and treatment

	1975‐1984		1985‐1994		1995‐2004		2005‐2009	
	No.	%	No.	%	No.	%	No.	%
**Men**								
All	1969		3273		4478		3859	
Histologic type(after imputation)								
UC	1827	92.8	3135	95.8	4296	95.9	3642	94.4
Non‐UC	142	7.2	138	4.2	182	4.1	217	5.6
Stage(after imputation)								
Localised	1462	74.3	2593	79.2	3573	79.8	3061	79.3
Regional	321	16.3	462	14.1	607	13.6	587	15.2
Distant	185	9.4	218	6.7	298	6.7	211	5.5
Age group								
0‐64	838	42.6	1384	42.3	1517	33.9	1027	26.6
65‐74	662	33.6	973	29.7	1573	35.1	1335	34.6
75‐99	469	23.8	916	28.0	1388	31.0	1497	38.8
Surgery								
Performed	1606	81.6	2822	86.2	3887	86.8	3467	89.8
Not performed	363	18.4	451	13.8	591	13.2	392	10.2
Radiotherapy								
Performed	226	11.5	244	7.5	316	7.1	201	5.2
Not performed	1743	88.5	3029	92.5	4162	92.9	3658	94.8
Chemotherapy								
Performed	1137	57.7	1735	53.0	1465	32.7	822	21.3
Not performed	832	42.3	1538	47.0	3013	67.3	3037	78.7
**Women**								
All	643		966		1221		1091	
Histologic type(after imputation)								
UC	564	87.7	880	91.1	1111	91.0	979	89.7
Non‐UC	79	12.3	86	8.9	110	9.0	112	10.3
Stage(after imputation)								
Localised	419	65.2	683	70.7	866	70.9	800	73.3
Regional	146	22.7	182	18.8	244	20.0	222	20.3
Distant	78	12.1	101	10.5	111	9.1	69	6.3
Age group								
0‐64	219	34.1	300	31.1	292	23.9	225	20.6
65‐74	231	35.9	291	30.1	348	28.5	298	27.3
75‐99	193	30.0	375	38.8	581	47.6	568	52.1
Surgery								
Performed	478	74.3	761	78.8	983	80.5	923	84.6
Not performed	165	25.7	205	21.2	238	19.5	168	15.4
Radiotherapy								
Performed	89	13.8	62	6.4	93	7.6	73	6.7
Not performed	554	86.2	904	93.6	1128	92.4	1018	93.3
Chemotherapy								
Performed	377	58.6	489	50.6	373	30.5	221	20.3
Not performed	266	41.4	477	49.4	848	69.5	870	79.7

Between 1975 and 2009 age‐standardized 5‐year net survival increased for both sexes (from 56.2% to 74.3% in men and from 49.1% to 69.2% in women) (Table 2) and a difference between sexes was evident during the whole period. The gap between sexes in 5‐year net survival of patients aged 75‐99 years widened (Table 2), probably because the number of elderly patients with bladder cancer is increasing.

**Table 2:** Five‐year net survival and its 95% confidence interval of bladder cancer patients in Osaka, Japan from 1975 to 2009

	1975‐1984		1985‐1994		1995‐2004		2005‐2009	
	%	95% CI	%	95% CI	%	95% CI	%	95% CI
**M** **en**								
All	56.8	53.8‐59.8	68.5	66.2‐70.9	73.1	71.2‐75	71.7	69.6‐73.9
Histological type(after imputation)								
UC	59.1	56‐62.1	70.2	67.8‐72.6	74.5	72.5‐76.4	73.3	71.1‐75.5
non‐UC	28.6	19‐38.1	31.6	22.5‐40.8	39.5	31.4‐47.7	44.6	35.7‐53.5
Stage(after imputation)								
Localised	73.5	70.0‐76.9	82.2	79.6‐84.7	86.5	84.5‐88.5	83.2	80.8‐85.7
Regional	14.4	9.7‐19.2	26.5	21.7‐31.4	27.5	23.2‐31.9	36.7	31.9‐41.4
Distant	7.3	3.1‐11.6	3.7	0.7‐6.7	8.2	4.7‐11.8	6.8	2.9‐10.6
Age group								
0‐64	67.7	64.1‐71.4	78.5	75.9‐81	81.6	79.4‐83.8	82.7	80‐85.4
65‐74	54.9	50.1‐59.8	69.6	65.8‐73.4	74.8	72‐77.6	74	71.1‐77
75‐99	41	33.8‐48.3	53.3	47.6‐58.9	61.9	57.5‐66.3	62.4	58.2‐66.7
Age‐standardized	56.2	51.3‐61.3	68.6	64.8‐72.4	73.9	70.9‐76.9	74.3	71.1‐77.5
Surgery								
Done	64.1	60.8‐67.3	75.7	73.2‐78.1	77.8	75.8‐79.7	76	73.8‐78.2
Not done	25.7	19.9‐31.5	25.9	20.2‐31.5	41.9	36.5‐47.3	33.9	27.7‐40.1
Radiotherapy								
Done	26.3	19.3‐33.3	28.6	21.7‐35.5	27.9	22‐33.9	28.7	20.7‐36.8
Not done	60.9	57.7‐64.1	71.8	69.4‐74.2	76.5	74.6‐78.5	74.1	72‐76.3
Chemotherapy								
Done	48.7	42.4‐55	61.7	56.1‐67.4	63.9	57.9‐69.9	54.8	47.1‐62.6
Not done	54.5	50‐58.9	67.4	64‐70.8	74.9	72.6‐77.3	75.1	72.7‐77.5
**Women**								
All	47.4	42.6‐52.3	57.5	53.5‐61.4	62.1	58.7‐65.5	61.6	57.9‐65.2
Histological type(after imputation)								
UC	50.9	45.5‐56.2	59.9	55.7‐64.1	66	62.4‐69.6	64.6	60.7‐68.5
non‐UC	26.1	16.1‐36.1	31.4	21‐41.7	24.1	15.8‐32.3	34.4	24.4‐44.4
Stage(after imputation)								
Localised	70.2	63.8‐76.5	78.5	73.9‐83.1	82.1	78.2‐85.9	77.1	72.8‐81.3
Regional	13.7	7.5‐19.8	14.3	8.7‐20	16.5	11.4‐21.5	26.4	19.9‐33
Distant	1.8	‐1‐4.6	5.1	0.7‐9.5	5.9	1.3‐10.5	2.8	‐1.1‐6.7
Age group								
0‐64	61.2	53.9‐68.6	66.8	60.9‐72.6	77.8	72.8‐82.7	83.2	78‐88.4
65‐74	47.2	39.8‐54.7	61.8	55.3‐68.3	70.8	65.5‐76.2	69.4	63.6‐75.1
75‐99	33.4	24.1‐42.7	47.2	39.9‐54.4	48.9	43.2‐54.5	48.7	43‐54.4
Age‐standardized	49.1	41.2‐57.1	59.7	53.2‐66.1	67.4	62.1‐72.6	69.2	63.7‐74.7
Surgery								
Done	60	54.2‐65.7	69.5	65.1‐74	72	68.2‐75.7	68.6	64.7‐72.6
Not done	14.1	8‐20.2	14.4	8.8‐19.9	21	15.1‐26.9	22.7	15.1‐30.2
Radiotherapy								
Done	30.2	18.8‐41.5	26	13.5‐38.5	22.8	13.2‐32.3	21.5	10.8‐32.3
Not done	50.3	45.1‐55.5	59.8	55.7‐63.9	65.3	61.8‐68.9	64.5	60.7‐68.3
Chemotherapy								
Done	58.5	54.6‐62.5	69.6	66.4‐72.8	69.1	65.9‐72.3	58.9	54.5‐63.3
Not done	45.5	38.1‐53	53	47.5‐58.5	61.1	57‐65.3	63.3	59.1‐67.4

Results from the univariate and flexible multivariate excess hazard model are shown in Table 3. The risk of death from bladder cancer is higher among women than men after adjusting for histologic type, stage, period at diagnosis, age group, and treatment (surgery, radiotherapy, and chemotherapy) (EHRs: 1.17; 95% CI: 1.10‐1.25). The following were identified as favorable prognostic factors: earlier stage, recent period at diagnosis, UC, younger age group, surgery performed, chemotherapy performed, and radiotherapy not performed.

**Table 3:** Excess hazard ratio of death within 5 years for bladder cancer patients diagnosed between 1975 and 2009 in Osaka, Japan using excess hazard model (n = 17 500)

Variables	Univariate		Multivariate	
	Excess hazard ratio	95% CI	Excess hazard ratio	95% CI
Sex				
Men	1.00		1.00	
Women	1.50	1.41‐1.60	1.17	1.10‐1.25
Period at diagnosis				
1975‐1984	1.00		1.00	
1985‐1994	0.69	0.63‐0.75	0.83	0.75‐0.91
1995‐2004	0.58	0.53‐0.63	0.65	0.59‐0.72
2005‐2009	0.57	0.52‐0.62	0.60	0.55‐0.66
Histologic type(after imputation)				
UC	1.00		1.00	
Non‐UC	3.06	2.77‐3.37	1.33	1.19‐1.49
Stage(after imputation)				
Localized	1.00		1.00	
Regional	6.94	6.48‐7.44	5.23	4.85‐5.64
Distant	15.76	14.40‐17.25	9.71	8.75‐10.78
Age group				
0‐64	1.00		1.00	
65‐74	1.61	1.50‐1.73	1.47	1.36‐1.59
75‐99	2.81	2.61‐3.02	2.20	2.03‐2.37
Surgery				
Performed	1.00		1.00	
Not performed	4.52	4.25‐4.81	2.12	1.98‐2.28
Radiotherapy				
Performed	1.00		1.00	
Not performed	0.31	0.29‐0.33	0.83	0.76‐0.91
Chemotherapy				
Performed	1.00		1.00	
Not performed	0.85	0.80‐0.90	1.15	1.08‐1.23

According to the stratified multivariate analysis by period at diagnosis, histologic type, stage, age group, and treatment (Table 4), the risk of death was higher among women with regional or distant stage cancer than men (EHRs: 1.26; 95% CI: 1.13‐1.40 for regional and EHRs: 1.22; 95% CI: 1.05‐1.41 for distant) but no significant difference was observed between the sexes at the localized stage. Significant difference between sexes was observed from 1985 to 2009 but not from 1975 to 1984. Overall, the prognosis for women was worse than that for men regardless of age group, stage, treatment type and whether or not treatment had been performed. Women, age 75‐99 with surgery not performed had a particularly poor prognosis compared to men (EHRs: 1.23; 95% CI: 1.12‐1.34 for age 75‐99 and EHRs: 1.27; 95% CI: 1.13‐1.42 for surgery not performed).

**Table 4:** Excess hazard ratio of death for bladder cancer within five years for females compared to males using excess hazard model, stratified by period at diagnosis, histological type, stage, age group, and treatment

Variables	Men	Women	95% CI
	EHRs	EHRs	
Period at diagnosis			
(adjusted for histologic type, stage, age group, surgery, radiotherapy, and chemotherapy)			
1975‐1984	1.00	1.02	0.88‐1.20
1985‐1994	1.00	1.22	1.08‐1.38
1995‐2004	1.00	1.15	1.03‐1.29
2005‐2009	1.00	1.29	1.13‐1.48
Histologic type			
(adjusted for period at diagnosis, stage, age group, surgery, radiotherapy, and chemotherapy)			
UC	1.00	1.18	1.10‐1.27
Non‐UC	1.00	1.16	0.97‐1.39
Stage			
(adjusted for period at diagnosis, histological type, age group, surgery, radiotherapy, and chemotherapy)			
Localized	1.00	1.03	0.93‐1.13
Regional	1.00	1.26	1.13‐1.40
Distant	1.00	1.22	1.05‐1.41
Age group			
(adjusted for period at diagnosis, histologic type, stage, surgery, radiotherapy, and chemotherapy)			
0‐64	1.00	1.12	0.97‐1.29
65‐74	1.00	1.10	0.97‐1.24
75‐99	1.00	1.23	1.12‐1.34
Surgery			
(adjusted for period at diagnosis, histologic type, stage, age group, radiotherapy, and chemotherapy)			
Performed	1.00	1.10	1.02‐1.19
Not performed	1.00	1.27	1.13‐1.42
Radiotherapy			
(adjusted for period at diagnosis, histologic type, stage, age group, surgery, and chemotherapy)			
Performed	1.00	1.08	0.92‐1.27
Not performed	1.00	1.19	1.11‐1.28
Chemotherapy			
(adjusted for period at diagnosis, histologic type, stage, age group, surgery, and radiotherapy)			
Performed	1.00	1.12	1.01‐1.24
Not performed	1.00	1.19	1.10‐1.30

**Figure 1:**
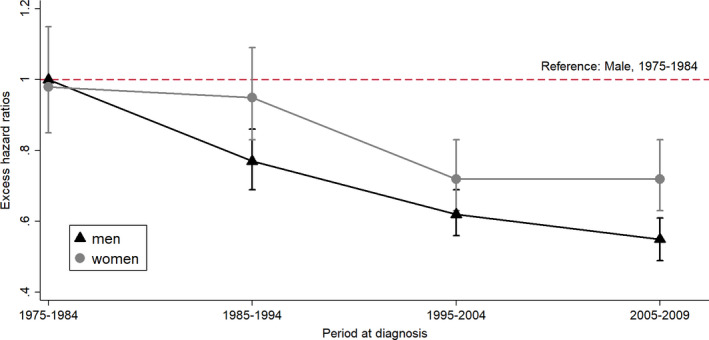
Trends in excess hazard ratios (EHRs) within 5 years in each period for both sexes when the male EHRs in 1975‐1984 is a reference. EHRs for interaction of sex and period at diagnosis were calculated adjusted for histologic type, stage, age group, and treatment

Results from the model, including the interaction between period at diagnosis and sex as a reference group of men who had been diagnosed from 1975 to 1984 (Interaction Model 1, Table 5), show a greater improvement in excess hazard of death within 5 years in men after adjusting for all covariates (Figure 1). Interaction Model 2, Table 5, which does not include histologic type or stage at diagnosis, shows the EHRs for women to be greater than those in Interaction Model 1. This indicates that stage and histology contribute to the improvement of EHR for women over time.

**Table 5:** Excess hazard ratio of death within five years for bladder cancer patients diagnosed between 1975 and 2009 in Osaka, Japan using excess hazard model including interaction between sex and period at diagnosis (n = 17,500)

Variables	Interaction Model 1		Interaction Model 2	
	Excess hazard ratio	95% CI	Excess hazard ratio	95% CI
Period at diagnosis (Men)				
1975‐1984	1.00		1.00	
1985‐1994	0.77	0.69‐0.86	0.74	0.67‐0.81
1995‐2004	0.62	0.56‐0.69	0.60	0.55‐0.66
2005‐2009	0.55	0.49‐0.61	0.60	0.54‐0.66
Period at diagnosis (Women)				
1975‐1984	0.98	0.85‐1.15	1.05	0.92‐1.21
1985‐1994	0.95	0.83‐1.09	0.96	0.87‐1.11
1995‐2004	0.72	0.63‐0.83	0.78	0.70‐0.88
2005‐2009	0.72	0.63‐0.83	0.80	0.70‐0.91
Histologic type (after imputation)				
UC	1.00		n/a	
Non‐UC	1.34	1.20‐1.50	n/a	
Stage (after imputation)				
Localized	1.00		n/a	
Regional	5.23	4.85‐5.64	n/a	
Distant	9.68	8.72‐10.74	n/a	
Age group				
0‐64	1.00		1.00	
65‐74	1.68	0.73‐3.86	1.60	1.49‐1.72
75‐99	2.53	1.00‐6.45	2.45	2.27‐2.63
Surgery				
Performed	1.00		1.00	
Not performed	2.13	1.98‐2.29	3.54	3.32‐3.77
Radiotherapy				
Performed	1.00		1.00	
Not performed	0.83	0.76‐0.91	0.48	0.44‐0.52
Chemotherapy				
Performed	1.00		1.00	
Not performed	1.15	1.08‐1.23	0.88	0.83‐0.94

## 4 DISCUSSION

### 4.1 Key findings

Women had poorer net survival than men throughout the study period. A high proportion of non‐UC, regional cancer, distant cancer, and age over 75 in women might explain the sex difference because the prognosis for these factors is significantly worse than that for UC, localized cancer, and age under 75 in our study. (Table 5) We found that sex difference increased proportionally with histologic type, stage, and age but that female sex was an unfavorable prognostic factor, even after adjusting for other covariates in the multivariate model: histologic type, stage, age, period at diagnosis, and treatment.

**Figure 2:**
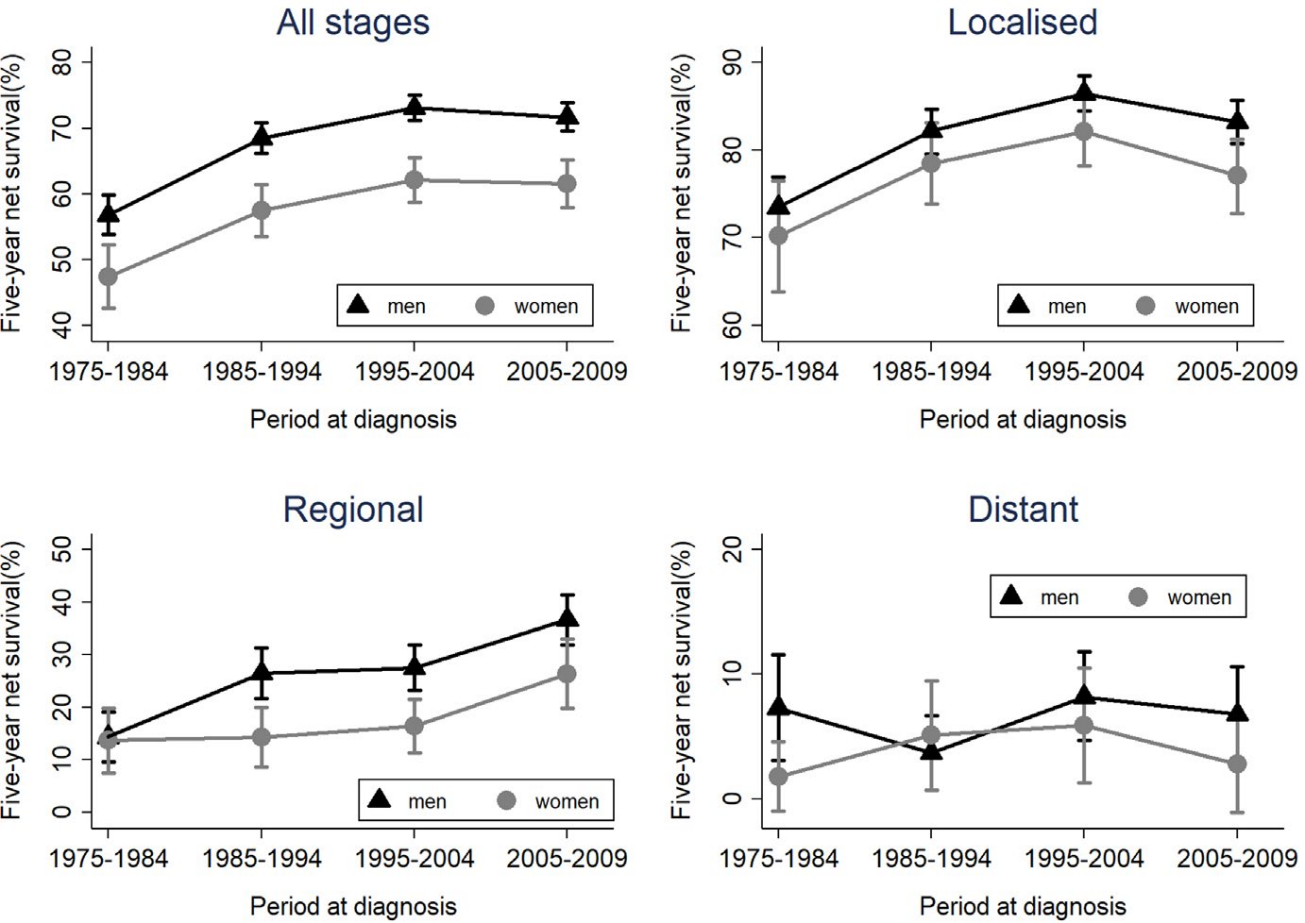
Five‐year net survival and its 95% confidence interval for bladder cancer patients in Osaka, Japan from 1975 to 2009 for each stage. Top left shows cancer at all stages, top right shows localized cancer, bottom left shows regional cancer, and bottom shows distant cancer

In terms of the long‐term trend, five‐year net survival for both men and women improved in the study period (Figure 2 and Table 2). By stage at diagnosis, localized cancer improved until 2004, regional cancer improved over the whole period, and distant cancer did not significantly improve over the whole period (Figure 2 and Table 2). The confidence intervals for the survival estimates in men and women overlap for all but regional cancer.

### 4.2 Possible explanation for the long‐term trend in five‐year net survival by stage

In 1976, Morales et al first demonstrated the antitumor effect of Bacille Calmette‐Guérin (BCG) in intravesical therapy for superficial bladder cancer.^22^ Soon thereafter, it was established worldwide as the standard treatment.^23^ Intravesical injection of BCG is also known to prevent recurrence after transurethral resection of bladder tumors (TURBT).^24^ The increase in five‐year survival in localized cancer is partly explained by the introduction of BCG and improvement of the methods of treatment.

The standard treatment for regional bladder cancer is a combination of total cystectomy and chemotherapy, and chemotherapy is the first‐line treatment for patients with distant metastases. The introduction of M‐VAC (methotrexate, vinblastine, doxorubicin, and cisplatin) therapy^25^ in 1985 as standard chemotherapy for bladder cancer led to a major change in the treatment of regional and distant bladder cancer. This could explain the improvement in five‐year net survival for regional cancer since 1985. However, "5‐year" net survival of distant cancer has not changed appreciably since the advent of M‐VAC, possibly because even with M‐VAC treatment, most patients die within two years,^26^ and thus make little impact on 5‐year net survival. Receiving radiotherapy was an unfavorable prognostic factor. Since the standard therapy for regional bladder cancer is radical cystectomy and chemotherapy, it is assumed that most of patients who receive radiation therapy have poor performance status or severe complications that make them unsuitable for surgery. A selection bias for radiation therapy could be a reason why not receiving radiation therapy is a favorable prognostic factor.

### 4.3 Factors which could explain sex difference in bladder cancer survival

Differences in anatomical structure, behavioral response to urinary problems and smoking rate between sexes would be possible explanations for the sex difference in bladder cancer survival.

There are a number of differences in anatomical structure between men and women that might contribute to the difference in net survival. It has been suggested that the thicker detrusor muscle in men,^27^ the less robust bladder neck in women,^28^ and the effects of growth of glandular prostatic tissue on angio‐lymphatic drainage in men^29^ may contribute to sex differences in the spread of bladder cancer. This may cause different survival rates within the same stage.

Delays in care seeking behavior among women in response to urinary problems and delays in referral to a urologist may also cause sex differences in survival. According to a study by Henning et al, women were more likely to be treated for urinary problems without further evaluation or referral to urology than men.^30^ A recent paper by Andreassen and colleagues focused on sex differences in survival for bladder cancer in Norway between 1997 and 2011.^31^ It found that only Norwegian women have a less favorable prognosis within the first 2 years after diagnosis. They concluded that parts of this discrepancy can be attributed to more severe initial diagnoses in women. Our data, which show women to have a lower proportion of localized cancer at diagnosis, support this theory. Moreover women with cystitis may delay case‐seeking; another previous study in Australia showed that a history of cystitis was significantly associated with poorer survival from bladder cancer and the association was present only in women.^32^ This evidence suggests that a delay in care seeking among women may contribute to their poorer prognosis. However, the authors did not suggest a reason for this association.

Difference in smoking behavior might also affect the bladder cancer survival gap between sexes. A previous study showed that never smokers had a 15% lower risk of death than current smokers for all‐cause mortality among patients with various cancers.^33^ In Japan, the prevalence of smoking in women was less than half that in men in 2010.^34^ Therefore, male hazard ratios (HRs) should be higher than female considering the effect of tobacco. However, our result shows the opposite. There are three possible reasons for this. First, in the previous study the increased HRs did not relate specifically to bladder cancer; it is possible that tobacco does not have a great effect on prognosis for bladder cancer patients. Although the topic is still under debate as shown in previous studies.^35^, ^36^, ^37^ Second, we did not have data on smoking behavior among bladder cancer patients and the sex difference in smoking behavior among bladder cancer patients might be small. Third, the risk of being a woman might outweigh the effect of tobacco. The Australian study cited above^32^ showed no significant difference in hazard ratio between non‐smokers and current smokers among bladder cancer patients. Further study is needed to determine whether the situation is the same in Japan by stratifying patients for smoking behavior.

Other factors such as differences in hormones, genes, socioeconomic status, and types of surgery received may contribute to the sex difference.^38^

### 4.4 Comparison with other studies

A previous study in Japan showed that women with localized or regional bladder cancer have a worse prognosis than men,^10^ while our study showed that women with regional or distant bladder cancer have a worse prognosis. This difference might be explained by the fact that our study only analyzed data from Osaka prefecture, while their data covered six prefectures. Moreover the difference in period at diagnosis might affect the result. We used data from 1975 to 2009, while their data only covered the period 1993 to 2006.

Previous work on sex differences in bladder cancer in Kanagawa, Japan showed that adjusted hazard ratio for cancer specific death of women to men was 1.39 (95% CI: 1.28‐1.52),^38^ which was higher than the EHRs based on our study results. The difference could be explained by controlling for stage and background mortality in our analysis.

The Australian study covering the period 1980 to 2003 yielded overall female HR of 1.11 (95% CI: 1.05‐1.18) after adjusting for age, socioeconomic status, country of birth, period at diagnosis, stage, and histology.^3^ This was similar to our result. As in our study, non‐UC, regional stage, distant stage, and elderly people have high hazards. In Australia, unlike Japan, there is little hazard reduction by period. Overall their results were consistent with ours.

The age‐standardized 5‐year survival rate for Japan (Men: 73.9(1995‐2004), 74.3(2005‐2009), Women: 67.4(1995‐2004), 69.2(2005‐2009)) was similar to or higher than that for Europe (65.2 to 73.0 depending on region.(2000‐2007) for both men and women). This result is considered to be a guarantee of the reliability of our analysis.

### 4.5 Limitations

We excluded cases that were DCO, according to the international standard method. However, as the percentage of DCO bladder cancer in the OCR has reduced during the study periods (from 13.5% in 1975‐1984, to 13.4% in 1985‐1994, 9.7% in 1995‐2004 and 8.5% in 2005‐2009), by excluding the DCO cases, we may have overestimated the net survival of bladder cancer and influenced trends in survival. Moreover the number of DCO cases for women was higher than for men. The higher number of DCO in women suggests that more dead cases were excluded from the female dataset. This might have influenced the higher survival rate in women compared to men.

The OCR data did not include details of care‐seeking behavior, history of cystitis, hormone levels of patients, genomes of patients, types of surgery, smoking status, or socioeconomic status. These factors could contribute to sex difference in bladder cancer survival. Moreover the OCR used SEER Summary Stage for cancer registration and therefore did not separate T1 and T2, but treated both as localized tumors, and did not separate T1‐2, N1‐3, M0 and T2‐3, N0‐3, M0, but treated both as regional disease. However, each TNM category has different treatment and different prognosis. For example, BCG immunotherapy is frequently performed in T1 patients, while radical cystectomy with chemotherapy is performed in T2 patients.^39^ This difference might affect mortality or sex difference.

### 4.6 Future recommendations

We need to understand the specific factors which result in sex difference in bladder cancer survival in order to inform governmental action for increasing female bladder cancer survival. However, the OCR is limited in that it does not have information about care seeking behavior, history of cystitis, patient hormone levels, patient genome levels, type of surgery, smoking status, or socioeconomic status. Therefore, it is important to create a linkage system between cancer registry data and medical history, genome information, smoking status, and socioeconomic status. Moreover, detailed information on TNM stage should be collected in the future to accurately assess the effect of different treatments.

With regard to trends, we expect to evaluate the effects of new treatments, like immune checkpoint inhibitors, by acquiring data from future periods and comparing them with our data, considering the possible background factors that may affect the results. In addition, because our results were limited to one city (Osaka), we need to compare them with results from multiple city analyses for confirmation. We will also acquire more detailed TNM information in the future as treatment strategies differ within the same SEER stage.

In conclusion, we examined sex differences in net survival in bladder cancer using data from Osaka's large‐scale, population‐based cancer registry. We showed the long‐term trend of five‐year net survival for each stage (Figure 2) and found that women had poorer net survival than men during the whole period. Although this may, in part, be due to sex difference in the proportions of histologic type, age, and stage, even after adjustment for these factors, female sex was an independent unfavorable prognostic factor. In order to improve bladder cancer survival among women, further studies are necessary to identify the specific factors which cause the sex difference.

## CONFLICT OF INTEREST

The authors have no conflicts of interest directly relevant to the content of this article.

## AUTHOR CONTRIBUTIONS

JA, YI, KF, and TN contributed to conception and design of study. YI, TM, and IM contributed to acquisition of data. JA, YI, KF, MN, TM, IM, TS, and TN contributed to analysis and/or interpretation of data. JA and YI contributed to drafting the manuscript. KF, MN, TM, IM, TS, and TN revising the manuscript critically for important intellectual content. JA, YI, KF, MN, TM, IM, TS, and TN contributed to approval of the version of the manuscript to be published.

## Supporting information

Table S1.

## Data Availability

The original individual data is only available to use for the researchers which were approved in the data usage committee of the Osaka Cancer Registry.
